# Treatment of vocal fold scarring with autologous bone marrow-derived human mesenchymal stromal cells—first phase I/II human clinical study: commentary to response

**DOI:** 10.1186/s13287-020-01748-x

**Published:** 2020-06-16

**Authors:** Stellan Hertegård, Katarina LeBlanc

**Affiliations:** 1 Department of Clinical Sciences and Intervention, Karolinska Institutet, Stockholm, Sweden; 2 Department of Otorhinolaryngology, Karolinska University Hospital Huddinge, S-141 86 Stockholm, Sweden; 3 Department of Laboratory Medicine, Karolinska Institutet, Huddinge, Sweden; 4 Department of Clinical Immunology and Transfusion Medicine, Karolinska University Hospital Huddinge, Stockholm, Sweden

**Keywords:** Vocal fold, Scarring, Hoarseness, Mesenchymal stromal cells, Fibrosis, Immunomodulation, Wound healing

## Abstract

We would like to thank for the interest for the article and comments in the response. We believe that cell therapy may offer a treatment for severe voice problems in patients with vocal fold scarring and MSC treatment is a valuable alternative.

## Commentary

The main aim of the study was safety aspects of the MSC treatment, and we found no side effects or complications after the treatment. All patients were followed for 1 year and subsequently for another 3–5 extra years. The 3 patients which were previously treated for laryngeal cancer (all at least 15 years before MSC treatment) have so far been followed between 5 and 7 years. We therefore think that the treatment is safe. Human BM-derived MSCs have never been reported to cause tumors in animals or humans. There are reports of in vitro transformation of human MSC, but these results were later shown to be the result of contamination by transformed cell lines in the cell lab [1–3]. Autologous (and allogeneic) MSCs have been administered, as local injection or intravenous infusion, to > 10,000 patients worldwide, without reported side effects. Long-term engraftment is low, and we avoid the risk for alloreactive reactions by using autologous cells. The harvest of the bone marrow from the iliac crest may result in local pain, but none of our patients complained about this. However, other sources of MSC may well be used as pointed out in the response. It is important to characterize the cells used in any clinical trials according to international guidelines.

The procedure with scar resection before MSC injection is discussed in the response. We chose this procedure because all our animal studies preceding the clinical human trial showed positive results regarding healing, inflammatory response, and functional result, both in “acute” damage model and after resecting present scar tissue [4–8]. It is true that scar resection alone may give some positive effects regarding vocal function in selected cases. There is however a clear risk for deteriorated voice and/or vocal fold function, and we were not willing to risk this, especially since this was the first study in humans. Also our previous animal studies showed significantly deteriorated viscoelastic properties and more scar formation, both in the acute damage model and after resection of present scar without the MSC treatment [4–6]. Another option is of course to have a control group where MSCs are injected without scar resection. Our results were less positive for patients with larger scar defects. We agree with the viewpoints in the response that these patients probably should be treated with a combination with MSC or other cell therapy (without scar resection) and a suitable scaffold which works well with the cells.

For the validated Swedish version of VHI30, a number of 13 points difference for total score is considered clinical relevant [9]. We would like to point out that only 3 patients were treated bilateral in our study although 9 had bilateral vocal fold damage. This may affect the results both for VHI and vocal fold parameters.

## Data Availability

The datasets generated and/or analyzed during the current study are not publicly available [due to data are kept protected following the routines of the Institution at Karolinska Institutet], but are available from the corresponding author on reasonable request.
